# Medicare reimbursement trends from 2000 to 2020 in head and neck surgical oncology

**DOI:** 10.1002/hed.27064

**Published:** 2022-04-13

**Authors:** Humzah A. Quereshy, Brooke A. Quinton, Claudia I. Cabrera, Shawn Li, Akina Tamaki, Nicole Fowler

**Affiliations:** ^1^ Department of Otolaryngology – Head and Neck Surgery University Hospitals Cleveland Medical Center Cleveland Ohio USA; ^2^ Case Western Reserve University School of Medicine Cleveland Ohio USA

**Keywords:** head and neck cancer, head and neck surgery, health economics, health policy, Medicare reimbursement

## Abstract

**Background:**

Considering limited data exploring reimbursement trends at the subspecialty level within head and neck surgical oncology, we sought to characterize these trends for head and neck‐specific codes from 2000 to 2020.

**Methods:**

Using the Centers for Medicare and Medicaid Services' Physician Fee Schedule Look‐Up Tool, reimbursement data, adjusted to 2020 U.S. dollars, for 37 head and neck surgical oncologic procedure codes were collected from 2000 to 2020.

**Results:**

From 2000 to 2020, despite gross reimbursement for all head and neck procedures increasing by 23.2%, when adjusted for inflation, physician reimbursement decreased by 19.4%. Only 4 of 37 examined codes increased in net reimbursement over the study period.

**Conclusion:**

Medicare reimbursement for the most common head and neck oncologic procedure codes decreased from 2000 to 2020. Further research is necessary to explore the implications of these trends on the delivery of patient care.

## 1 INTRODUCTION

Medicare Part B pays for physician services, which include procedures, office visits, anesthesia services, and other diagnostic codes, based on the Medicare Physician Fee Schedule, or the complete listing of fees used by Center for Medicare and Medicaid Services (CMS) to pay health care providers. For any given procedure or service, as defined by their designated Current Procedural Terminology (CPT) code, payment is dictated by three factors: (1) the Relative Value Units (RVU), (2) The Geographic Practice Cost Index, which adjusts for geographic variation in cost of care, and (3) a conversion factor to convert RVUs to dollars.^1^ This conversion factor changes on an annual basis in accordance with a statutory formula but continue to fluctuate irregularly. For example, over the last 20 years, the greatest decrease in conversion factor was noted in 2011 (−7.9%), while the greatest increase was just a few years later in 2014 at +5.3%.^2^ In 2021, the CMS conversion factor is $34.8931 per RVU.

Each code's allocated RVUs have three components: (1) work time and intensity, (2) practice expense and overhead, and (3) malpractice insurance.^1^ Using this algorithm, each code within each locality is dictated a certain reimbursement by the Centers for Medicare and Medicaid Services, which is used to pay providers on a Fee‐for‐Service basis.

Given the complexity and the vague nature of RVU allocation to certain CPT codes, there is a great deal of uncertainty providers and hospitals face when it comes to reimbursement. Several studies have documented flaws in CMS reimbursement methodologies, especially given the tedious process of revaluation of CPT codes, often taking years of questioning and evaluation before going into effect.^3^, ^4^ Accordingly, inadequate compensation for certain procedure codes may risk disincentivizing the provision of certain services that are critical to head and neck surgical practices. In this way, there is a growing need to characterize and understand reimbursement for otolaryngologic codes to appraise the valuation system and identify opportunities for change.

Reimbursement trend analysis is not new to otolaryngology. Specialty‐level analysis of 2017 Medicare Part B data suggested that otolaryngologic operative procedures were compensated significantly less than other surgical subspecialties.^5^ Given the high variance across otolaryngology, namely across subspecialties, more granular analysis has been to characterize where the disparities lie. Dominguez et al. assessed the trends of the top 20 most commonly billed otolaryngology codes, a vast majority of which were office‐based CPT codes, and found significant depreciation in the value over a 20‐year study period.^6^ Schartz and McCool in 2021 conducted a similar analysis of selected otologic codes and shared similar findings.^7^

While these studies examine important questions on reimbursement for otolaryngologic procedures, there lacks an analysis of reimbursement trends for the most common head and neck surgical oncologic codes. A cross‐sectional analysis of 2018 CMS data by Kondamuri et al. suggest that ablative head and neck surgical codes are among the middle of the pack for inpatient payment rates and the lowest ambulatory payment rate, when compared to other otolaryngologic subspecialties.^8^ An investigation exploring reimbursement trends to these procedure codes will add to the increasing insight into the unique incentives surrounding the provision of cancer care in otolaryngology and could springboard important discussions on reimbursement reform within the subspecialty. In this study, we aimed to compare temporal trends in CMS reimbursement for the most commonly billed surgical procedures within head and neck surgical oncology from 2000 to 2020.

## 2 MATERIALS AND METHODS

This study was determined as non‐human research from the Institutional Review Board at University Hospitals Cleveland Medical Center. Two head and neck surgeons at our institution (AT, NF) identified the most commonly billed CPT codes within the institution's practice (Table 1).

**TABLE 1:** Most commonly billed current procedural terminology (CPT) codes with corresponding procedure in head and neck surgical oncology

CPT code	Code description
15732	Muscle, myocutaneous, or fasciocutaneous flap; head and neck
15756	Muscle or myocutaneous free flap; microvascular transfer
15757	Free skin flap; microvascular transfer
20969	Free osteocutaneous flap with microvascular anastomosis
21044	Excision of malignant tumor of mandible
21045	Excision of malignant tumor of mandible; radical resection
21557	Radical resection of tumor of soft tissue of neck or anterior thorax; less than 5 cm
21558	Radical resection of tumor of soft tissue of neck or anterior thorax; 5 cm or greater
31225	Maxillectomy; without orbital exenteration
31230	Maxillectomy; with orbital exenteration
31360	Laryngectomy, total; without radical neck dissection
31365	Laryngectomy, total; with radical neck dissection
31367	Laryngectomy, subtotal supraglottic; without radical neck dissection
31390	Pharyngolaryngectomy with radical neck dissection
38720	Radical lymphadenectomy
38724	Cervical lymphadenectomy
41120	Glossectomy; less than one‐half tongue
41130	Glossectomy; hemiglossectomy
41135	Glossectomy; partial, with unilateral radical neck dissection
41140	Glossectomy; complete or total with or without tracheostomy without radical neck dissection
41145	Glossectomy; complete or total with or without tracheostomy with unilateral radical neck dissection
41150	Glossectomy; composite procedure with floor of mouth and mandibular resection without radical neck dissection
41155	Glossectomy; composite procedure with floor of mouth resection, mandibular resection, and radical neck dissection
42415	Excision of parotid tumor or parotid gland; lateral lobe, with dissection and preservation of facial nerve
42420	Excision of parotid tumor or parotid gland; total, with dissection and preservation of facial nerve
42425	Excision of parotid tumor or parotid gland; total, en bloc removal with sacrifice of facial nerve
42440	Excision of submandibular gland
42450	Excision of sublingual gland
60210	Partial thyroid lobectomy, unilateral; with or without isthmusectomy
60220	Total thyroid lobectomy, unilateral; with or without isthmusectomy
60225	Total thyroid lobectomy, unilateral; with contralateral subtotal lobectomy, including isthmusectomy
60240	Thyroidectomy, total or complete
60252	Thyroidectomy, total or subtotal for malignancy; with limited neck dissection
60260	Thyroidectomy, removal of all remaining thyroid tissue following previous removal of a portion of thyroid
60271	Thyroidectomy, including substernal thyroid; cervical approach
60500	Parathyroidectomy or exploration of parathyroid(s)
60605	Excision of carotid body tumor; with excision of carotid artery

Reimbursement data for these codes were obtained from CMS using the Physician Fee Schedule Look‐Up Tool.^9^ The facility reimbursement value was collected for each CPT code from 2000 to 2020. In 2007, CMS implemented a “National Payment Amount” as the reimbursement rate for a given code before the Geographic Practice Cost Index (GPCI). For all codes from 2000 to 2006, the rates for all CMS localities were averaged as a proxy for the National Payment Amount.

For each procedure, the unadjusted total and annual percent change in reimbursement was calculated and averaged. To standardize amounts over time, the reimbursement rate for each CPT code was adjusted for inflation to 2020 U.S. dollars over the study period using changes in the Consumer Price Index (CPI). The latest available data for the CPI was obtained from the U.S. Department of Labor's Bureau of Labor Statistics. This adjusted data was then used to analyze trends in reimbursement in 2020 dollars for the most frequently billed procedures in head and neck surgical oncology. Finally, *R*‐squared values were calculated to confirm the accuracy of time as a predictor of adjusted annual reimbursement decrease. A subanalysis was performed by anatomic region per procedure to determine if there exist any trends in reimbursement based on surgical site. Analyses were made in Microsoft Excel.

## 3 RESULTS

**FIGURE 1:**
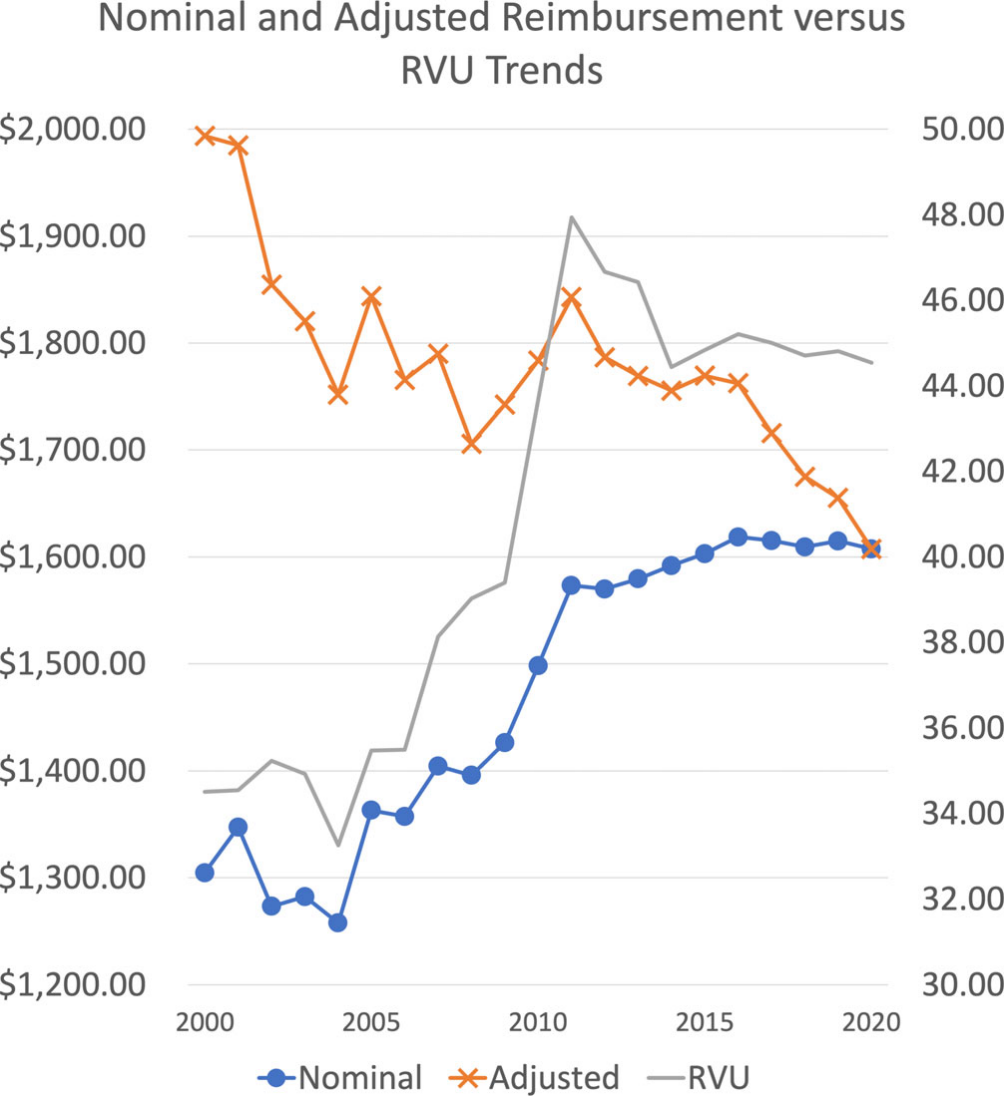
Nominal (blue line) and adjusted (orange line) facility reimbursement over time in dollars. The gray line shows corresponding relative value units (RVUs) over time [Color figure can be viewed at wileyonlinelibrary.com]

Head and neck surgical oncology CPT codes have increased in nominal reimbursement by 23.2% since 2000. However, when adjusted for inflation, reimbursement decreased by 19.4% over the study period. Conversely, RVUs allocated per procedure have gone up steadily over time. Furthermore, our results revealed that over time, procedures are reimbursed less, but are valued at higher RVUs (Figure 1).

**FIGURE 2:**
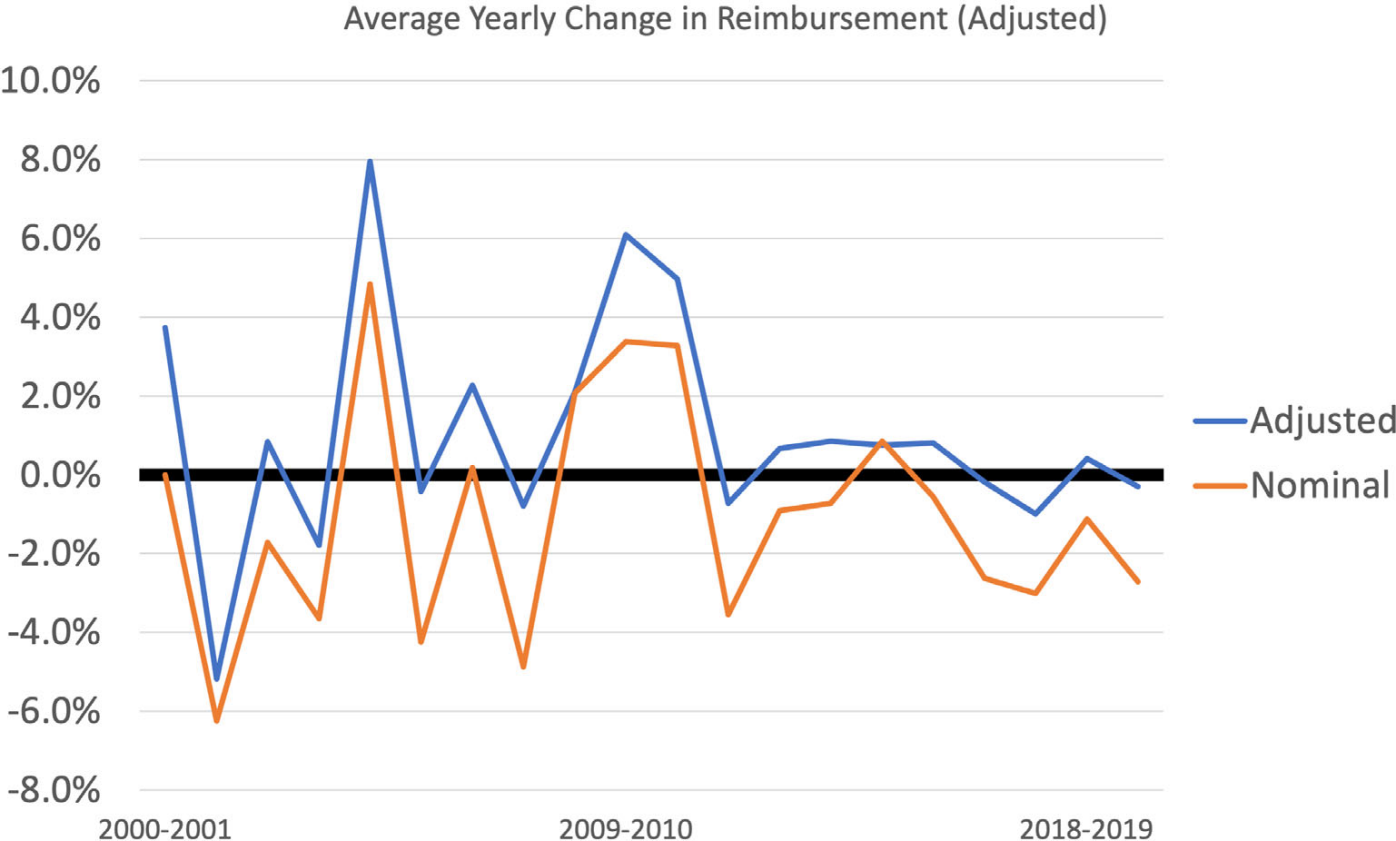
Average percent change in adjusted and nominal facility reimbursement annually from 2000 to 2020 [Color figure can be viewed at wileyonlinelibrary.com]

In addition, our findings suggest that the net negative effects on facility reimbursement are consistent annually with an approximate 1.1% decrease per year (Figure 2).

**FIGURE 3:**
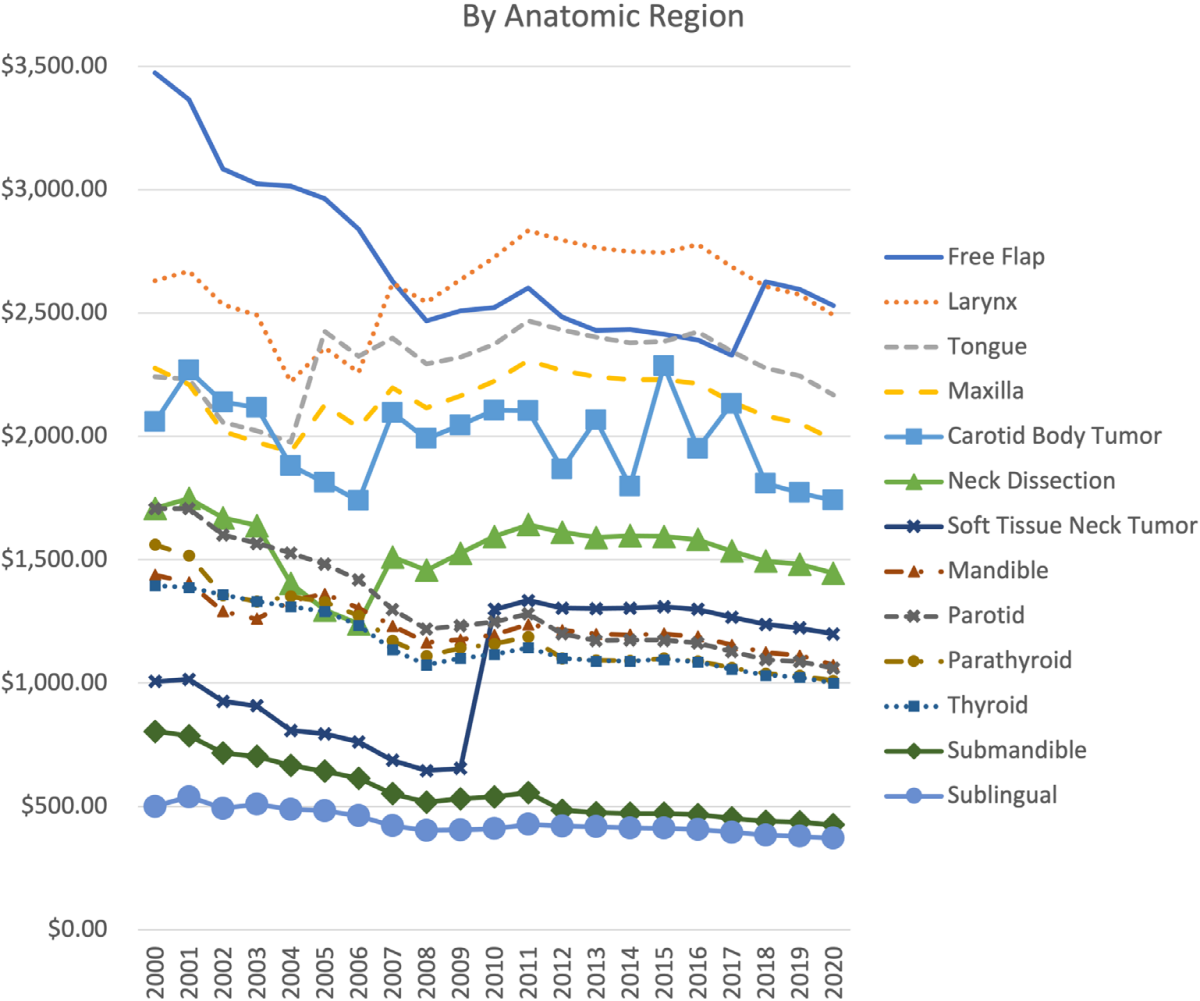
Trends in facility reimbursement by anatomic region or procedure type (free flap reconstruction, larynx, tongue, maxilla, carotid body tumor resection, neck dissection, soft tissue neck tumor excision, mandible, parotid, parathyroid, thyroid, submandibular gland, and sublingual gland) [Color figure can be viewed at wileyonlinelibrary.com]

**FIGURE 4:**
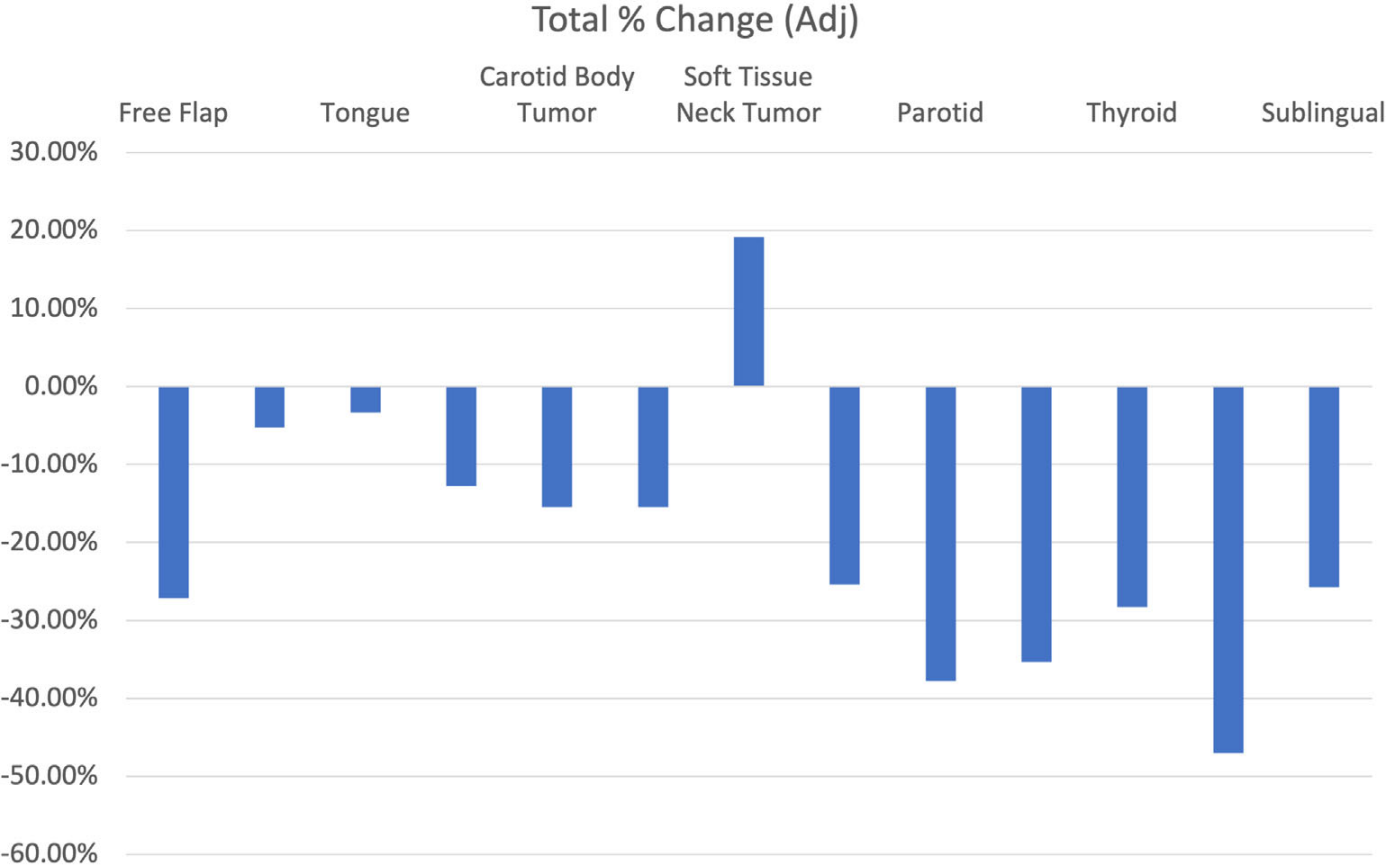
Total percent change in facility reimbursement by anatomic region or procedure type adjusted for inflation over the study period [Color figure can be viewed at wileyonlinelibrary.com]

We were able to characterize reimbursement changes on a per‐procedure basis to determine variability between procedure types (*p* < 0.001). These results showed that nearly all procedure types experienced a decrease in reimbursement when adjusted for inflation (Figures 3 and 4).

The greatest mean decrease was seen in CPT code 42440 (Excision of submandibular gland) at −47.0%. Only four procedures increased in reimbursement over the study period. The greatest mean increase was seen in CPT code 41130 (glossectomy; hemiglossectomy) at 14.1%.

For each code, *R*‐squared values were obtained to assess the strength of the correlation between time and changes in reimbursement. The range for this set of *R*‐squared values was 0.00–0.92. The average *R*‐squared value for this analysis was 0.51 indicating a moderate correlation between time and a decrease in compensation (Figure 3).

## 4 DISCUSSION

This study reveals that average Medicare reimbursement rates for the most frequently billed procedures in head and neck surgical oncology at our institution decreased by approximately 19% from 2000 to 2020 when adjusting for inflation, despite an increase in nominal, or unadjusted, reimbursement by 23% for these same codes. In comparison to other otolaryngology codes, head and neck codes revealed a less dramatic decrease in physician reimbursement, as demonstrated by the 38% decrease in reimbursement for the top 20 billed codes in otolaryngology as shown by Dominguez et al.^6^ In addition, our study revealed that, while all codes experienced some degree of reduction in adjusted reimbursement, certain anatomic sites were disproportionately affected by these reductions over time. Finally, while we found a mean annual reimbursement reduction of 1.1% per year while adjusting for inflation, it is interesting and important to note that there were significant fluctuations in reimbursement each year. This suggests that, while, on net, adjusted reimbursement is downtrending, market‐based effects contributing to fee schedule changes are consistently changing and can explain year‐to‐year variability. Understanding these reimbursement trends in the context of a changing health care market is necessary to ensure appropriate support for future head and neck surgical oncology physicians and their patients.

The implications of decreased reimbursement rates over time for many of the most common procedures in head and neck surgical oncology are significant. For example, federally funded health insurance programs such as Medicare and Medicaid play an increasingly important role in health care reimbursement, as the decisions of private payers are largely influenced by the covered services and reimbursement rates set in place by CMS.^10^ Furthermore, the trend towards physician consolidation into large hospital centers increasingly obliges providers to accept CMS insurance coverage.^11^, ^12^ In this way, the steady decline of Medicare fee schedule rates for head and neck surgical oncology CPT codes has the potential to impact the success of head and neck surgical oncology practices, and ultimately alter global access to care. Specifically, the National Bureau of Economic Research published a report in 2019 concluding that changing financial incentives for providers can improve access to care for Medicaid recipients—for every $10 increase in reimbursement, Medicaid recipients were 1.4% more likely to report a doctor visit.^13^

Another important consideration for future studies includes how reimbursements within specific subspecialties of otolaryngology are impacted by their unique patient demographics and protocols for CMS reimbursement of particular procedures. For example, a recent study analyzing predictors of reimbursement for oral‐maxillofacial surgery services in the Medicare population found that academic and cancer surgeons were independently reimbursed less than other surgeons due to CMS negotiations, indicating that cancer cases may be more vulnerable due to negotiations favoring lower payout rates.^14^ Alternatively, given the prevalence and clinical implications of head and neck cancer, surgical oncology codes may undergo more frequent revaluation by CMS compared to CPT codes treating less morbid disease processes. In particular, the reductions in head and neck code reimbursement paled in comparison to that of the top 20 billed otolaryngology procedures, as noted in Dominguez et al.^6^ Many of the most frequently billed codes that saw the greatest reduction in adjusted reimbursement were endoscopic rhinology procedures, which may reflect improved technology and a learning curve that has ultimately resulted in faster operative times and more efficient operations.

Existing strategies to improve physician reimbursement rates include merit‐based incentive programs and alternative payment methods such as episode‐based payments and Accountable Care Organizations (ACOs).^15^ While these strategies aim to enact positive change in physician reimbursement through incentivizing high‐quality care while minimizing spending and have been implemented on small scales, higher‐volume trials and advancements are necessary before an appropriate reimbursement model is found.^16^ As this is one of the first studies to analyze the reimbursement rates within head and neck surgical oncology. There is a significant need for further research and advocacy within the field of otolaryngology regarding this topic to ensure sustainability and growth of future practices.

There are limitations to this study. Medicare reimbursement data alone was utilized in this analysis, and therefore it does not capture physician reimbursement for all head and neck surgical oncology procedures as some patients participate in private health insurance plans which are not accounted for. Future studies could evaluate differences in reimbursement between private practice compared with academic centers to achieve a more complete view of reimbursement for physicians within the field.

## 5 CONCLUSION

From 2000 to 2020, head and neck surgical oncological CPT codes have experienced a steady decline in inflation‐adjusted reimbursement, while RVU valuation of these codes have progressively risen. These trends parallel a greater trend across otolaryngology towards diminished reimbursement for providers; however, nuances within head and neck surgical oncology must be explored to further explain differences between head and neck and other subspecialties within otolaryngology. Considering political pressures to reduce reimbursement for providers, novel payment strategies must be implemented to improve physician reimbursement and sustain the growth of our specialty.

## Data Availability

The data that support the findings of this study are available from the corresponding author upon reasonable request.
